# Dr. Younes Karimi (1929-2008), the Prominent Iranian Physician in the Field of Plague and Other Infectious Diseases

**Published:** 2019-01

**Authors:** Sepideh Mahdavi, Alzira MP de Almeida, Ehsan Mostafavi

**Affiliations:** 1 Department of Epidemiology, School of Public Health, Shahroud University of Medical Sciences, Shahroud, Iran; 2 Department of Microbiology, Institute Aggeu Magalhaes-Fiocruz PE, Recife, PE, Brazil; 3 Department of Epidemiology and Biostatistics, Research Centre for Emerging and Reemerging infectious diseases, Pasteur Institute of Iran, Tehran, Iran

Dr. Younes Karimi was born in 1929 in Lotfabad Dargaz, Razavi Khorasan Province, where he accomplished the primary education. He received his diploma in Mashhad, northeast of Iran and followed his medical education and specialty in Infectious Diseases and Tropical Medicine at the University of Tehran by presenting his Ph.D. thesis entitled “Dried smallpox vaccine” in 1975. He also attended microbiology and immunology courses at the Institute Pasteur of Paris. Returning to Iran, he was engaged at Pasteur Institute of Iran and devoted his scientific career for researching on infectious diseases and the control of these diseases in Iran. He was also the head of the Department of Epidemiology and the Deputy Director of Pasteur Institute of Iran[1]. He was a hard-working person with scientific prestige and dignified character and a man of action interested in the field activities.

**Fig. 1:**
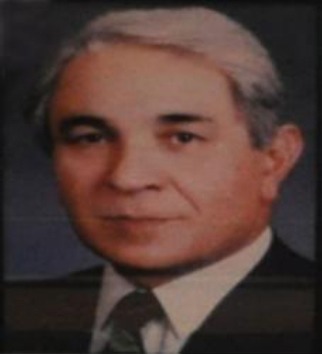
Dr. Younes Karimi (1929-2008).

Dr. Karimi’s major scientific research was in the field of plague. Furthermore, he conducted a number of researches on other infectious diseases such as tularemia, relapsing fever, hemorrhagic purpura, hepatitis B, and brucellosis. In this paper, his scientific career is reviewed and discussed

## Plague

The importance of plague disease in the Kurdistan plague foci, west of Iran, discovered in 1946, motivated Dr. Karimi along with Dr. Marcel Baltazard, Mansour Shamsa, Abdullah Habibi, Mahmoud Bahmanyar, Mirza Aqa Eftekhari, Abdul Rahman Farhang-Azad, Buick Seyyedian, Hooshang Majd Teimoury, and a group of trained technicians conducting scientific epidemiological studies in Kurdistan for several years. Uninterrupted efforts of the Pasteur Institute of Iran Plague Research Team based on the Research Center of Akanlu in the west of Iran, on the border of the provinces of Hamadan, Kurdistan, and Zanjan, made this center as one of the most famous centers for the plague reference internationally[2,3]. These groups controlled plague epidemics in the west and northwest of Iran, identified the plague-resistant and -susceptible rodents and the outstanding role of the *Meriones* in the plague maintenance and transmission[2,4,5]. Moreover, Dr. Karimi and a group of plague experts wrote the first instructions on laboratory diagnosis of plague bacillus[6]. Dr. Karimi also introduced a rapid laboratory diagnosis for plague[7]. During plague outbreaks in Kurdistan and Azerbaijan (1946-1965), numerous plague patients were rescued by expeditionary groups of Pasteur Institute of Iran[8]. In those years, the combination of field survey and laboratory tests provided a significant contribution to effective epidemiological control actions, resulting in extensive research hypothesis. Through field studies, Baltazard and his Iranian colleagues, including Dr. Karimi and Shamsa identified the rodents plague-endemic area in Kurdistan. They did not limit their research on plague microbiology, but they determined the wild reservoirs and vectors of the disease in nature. Their efforts ended up in a major finding: among the four *Meriones* species, two (*Meriones persicus* and *Meriones libycus*) were plague resistant and two *(Meriones tristrami and Meriones vinogradovi)* were plague susceptible. For the first time, they established the hypothesis that the resistant species were the real plague hosts instead of the susceptible ones. Nowadays, this hypothesis is fully accepted[9]

**Fig. 2:**
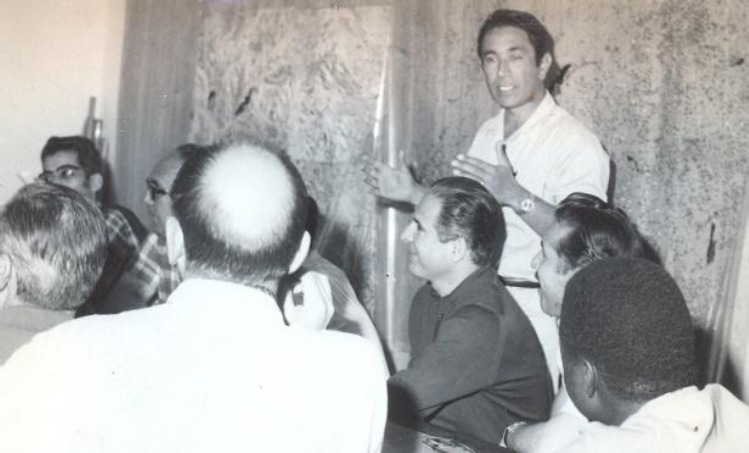
Dr. Younes Karimi speaking at the international workshop of the World Health Organization on plague, 1970, the Research Centre for Emerging and Reemerging infectious Diseases (Plague Centre of the Pasteur Institute of Iran), Akanlu Kabudar Ahang, Hamadan.

In addition, for the first time, Dr. Karimi reported the presence of *tripectinata spinellosa* flea on *Meriones persicus* specimens. This flea species is the only kind in Asia and for the first time, it was reported from Letian Dam at 25 km of the northeast of Tehran[10].

Dr. Karimi devoted his life to research on the plague in different parts of Iran, including Kurdistan and Azerbaijan Provinces[1,11]. His seminal finding was the survival of the plague bacilli in soil of dead rodent burrows. This finding was introduced as “burrowing plague” to the world scientific community, which could explain the survival of plague in nature. He observed that the plague bacteria could survive in soil for several years[12,13] and assumed this was the reason of the persistence of plague bacteria in the soil[14]. Furthermore, with Drs. Baltazard and Henri H. Mollaret, plague reemergence among rodents after three to five years of incubation period in soil, was experimentally demonstrated[15, 16].

Dr. Karimi detected the susceptibly of some *Escherichia coli* strains to the plague bacteriophage and described a proper method for rapid plague diagnostic[17]. Moreover, he remarked that the foxes feeding on alive or dead infected rats produce *Y. pestis* antibodies and assumed that testing the sera of foxes for the presence of plague antibodies would serve to uncover wild plague circulation in nature[18].

In 1978, a new focus of plague was reported by Dr. Karimi and his colleagues in Sarab area in East Azerbaijan Province in northwestern Iran[19]. The success of Pasteur Institute of Iran on plague research and control drew the WHO attention and led to the cooperation of Iranian experts in international activities on plague. Dr. Karimi conducted his research in different countries such as Brazil[20-25], Burma[26,27], and Congo[22] and brought his experiences to these countries.

In Brazil, as a prominent plague expert working for the WHO, he conducted three successful missions (March to November 1967, April 1969 to March 1970, and from May to July 1971). During these missions, under the aegis of Baltazard and with the Brazilian staff, a broad research program was developed in the Chapada do Araripe plague focus located in the state of Pernambuco, northeast of Brazil[28]. Their studies allowed to determine the rodent species involved in plague epizootiology[26], susceptibility to *Y. pestis* of several rodent species[21], and the vector ability of the rodent fleas (*Polygenis bolshi jordani* and *P. tripus*), resistance of the “rat” flea (*Xenopsylla cheopis*), and the “human flea” (*Pulex irritans*) to organochlorine insecticides[20], the isolation and characterization of a number of *Y. pestis* strains[24], as well as development of a new methodology for rapid plague diagnostic. These findings contributed to structuring a novel national plague surveillance program[28].

**Fig. 3:**
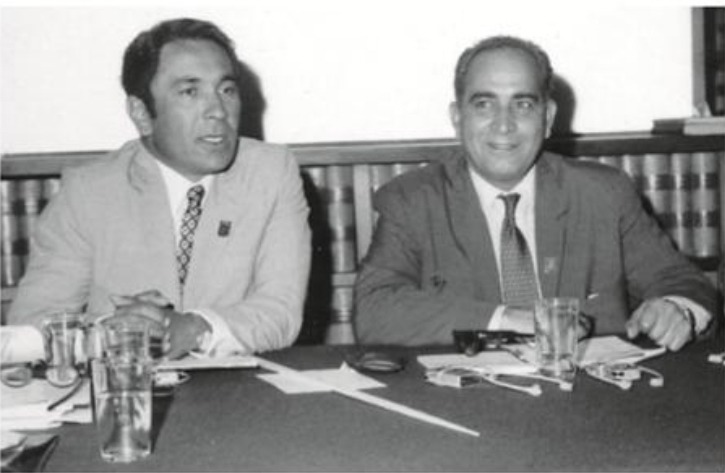
From the right to left: Dr. Mahmoud Bahmanyar and Dr. Younes Karimi at an international meeting on plague at the Pasteur Institute of Iran, 1970.

In addition, studies on the biology and parasites of the rodents and other small mammals were carried out[29,30], and a new marsupial was named *Marmosa karimi* in honor of Dr. Karimi[31]. Dr. Karimi was a prominent and famous physician among plague specialists worldwide owing to his expanded research on plague. He was a source of pride for Iranian scientists and for the Pasteur Institute of Iran.

## Study on other infectious diseases

**Tularemia:** In 1981, Dr. Karimi identified and reported the first human case of tularemia in Marivan, Kurdistan Province[32].

**Relapsing fever:** In 1976, Dr. Karimi discovered and described a new species of *Borrelia* in Ardebil, northwest of Iran, and named it *Borrelia baltazardi* in honor of his beloved director, Dr. Baltazard[33]. He developed a new protocol for relapsing fever control in 1980[34] in which the *Borrelia* spp. was eliminated from the ticks feeding on the blood of domestic animals receiving tetracycline[35].

**Rabies:** Another remarkable contribution was discovering wild rabies foci, and for the first time he reported the role of foxes in rabies epidemiology in Iran. Through serum samples testing, rabies antibodies were detected in 13.5% of 193 foxes[36]. The brilliant results of the Pasteur Institute of Iran staff using the combined administration of antiserum and vaccine post exposure the bites of rabid animals led the WHO to recommend the antiserum and vaccine combination to treat and cure the patients. During 13 years, the use of this rabies complex treatment resulted in decreasing the mortality rate from 12% to 1.5%[37].

**Hemorrhagic purpura:** As results of his investigations in East Azerbaijan Province on a kind of bleeding disorder characterized by a reduction of blood platelets and seasonal activity, he introduced the possible role of a tick-borne virus named Hemorrhagic purpura[32].

**Brucellosis:** Dr. Karimi and his colleagues conducted a study on 150 patients of Malta fever disease and developed several protocols to treat the patients[38].

**Hepatitis B:** He conducted investigations on Hepatitis B (Australian antigen), but the results have not been published.

Some of his other scientific papers were published in different Iranian journals such as Daneshmand[39] and Sokhane Pezeshki[40].

**Fig. 4:**
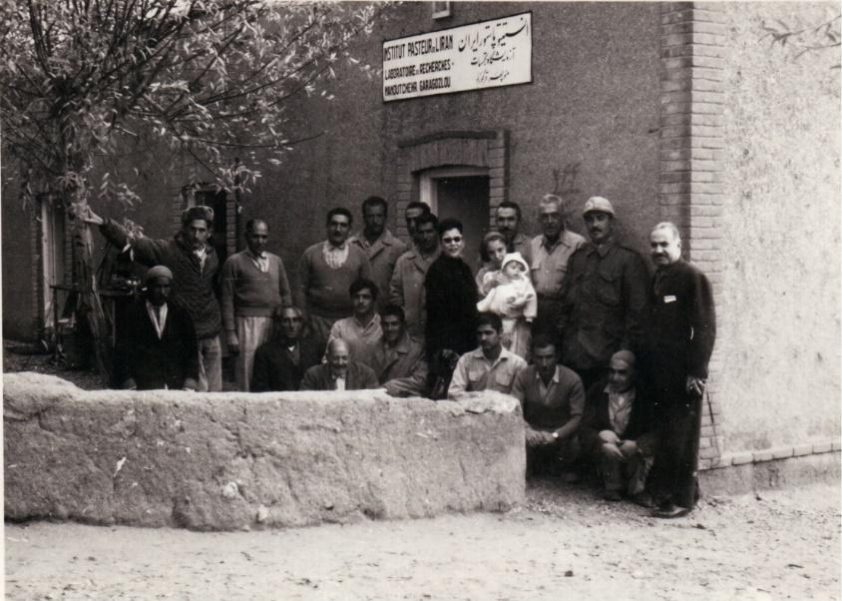
From right to left: Mr. Nabavi, Dr. Younes Karimi, Dr. Marcel Baltazard, Ali Ghahramani, Dr. Kellen’s wife, Mohammad Hanifi, Feizollah Salarkia, Hamid Salarkia, Salman Mesbah, Mousa Hakimi, and Dr. Kellen; the Research Centre for Emerging and Reemerging infectious Diseases (Plague Centre of the Pasteur Institute of Iran), Akanlu, Kabudar Ahang, Hamadan, 1957.

## Books

In addition to a number of international papers in the field of plague, Dr. Karimi published several books. In the book entitled “Plague and Its Epidemiology”, published by the Pasteur Institute of Iran in 1977, he presented the history of plague disease in Iran and in the world. Furthermore, the plague microbiology, geographical distribution, diagnosis, and epidemiology are described. This book contains valuable information on the rodent species maintaining *Y. pestis* in nature and transmitting the infection to humans in Iran[2].

Dr. Karimi and colleagues wrote the book “An Illustrated Key for Flea of Iran” in 1980. In this book, an overview of different flea and host species as well as their geographical distribution in Iran is presented[41]. The book “Relapsing Fever and Its Epidemiology”, published in 1982, describes the disease, clinical symptoms, and its epidemiology in Iran and the world. Furthermore, various *Borrelia* species are described[42].

In 1984, Dr. Karimi and his colleagues also wrote the book “Toxoplasmosis, Tularemia, and Listeriosis”. In this book, exhaustive recordings concerning various aspects of microbiology, epidemiology, and diagnosis of toxoplasmosis, tularemia, and listeriosis are presented[43].

Eventually, Dr. Karimi’s last book entitled “The Role of the Fox and Its Prey (in relation to medical issues)” was published in 1986. Considering the important role of foxes as the source of rabies virus and their role in the transmission of plague in Iran, this book provided comprehensive information about this animal’s physical traits, lifestyle, and the way of hunting[44].

**Fig. 5:**
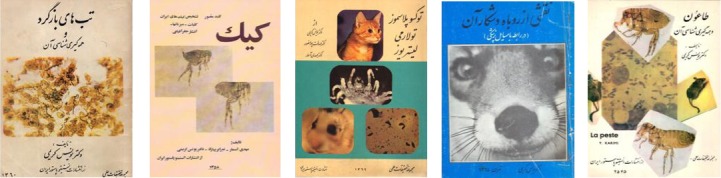
Cover of Dr. Younes Karimi’s books.

**Fig. 6:**
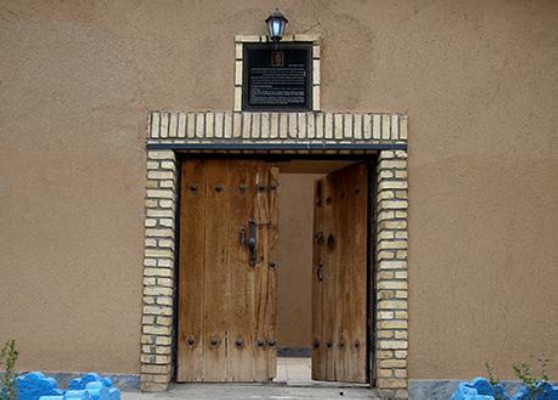
The monument of Dr. Younes Karimi at Akanlu Plague Research Center of the Pasteur Institute of Iran, Akanlu, Kabudar Ahang, Hamadan.

Dr. Karimi passed away in 2008 at the age of 79. In order to thank his services, a commemoration was held at Pasteur Institute of Iran[45].


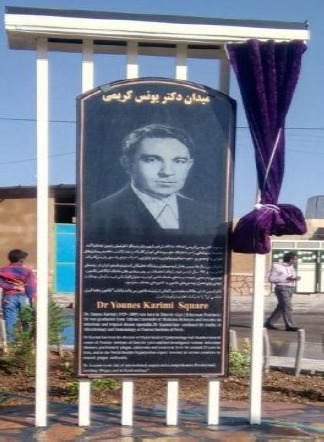


In 2013, in order to thank his services in the Akanlu Plague Research Center, in the Research Centre for Emerging and Reemerging Diseases, a renovated building was named after him and in 2016, a square in the entrance of Akanlu was also named after him.
